# Supplementary material for: Biomass Sorghum (Sorghum bicolor) Agronomic Response to Melanaphis sorghi (Hemiptera: Aphididae) Infestation and Silicon Application
Source: Insects. 2025 May 27;16(6):566. doi: 10.3390/insects16060566 (PMC12192828; doi:10.3390/insects16060566)
Supplement: Supplementary file 1 [file insects-16-00566-s001.zip › insects-3618812-supplementary.pdf]

Supplementary Materials:

Table S1. Summary of analysis of variance (ANOVA) and probability values (*P*) for Infestation and Damage scores under different silicon doses.

Source of Variation	Degrees of Freedom	Infestation (*P*-value)	Damage (*P*-value)
DAE	6	<0.001	<0.001
Silicon dose	3	<0.001	<0.001
Dose × DAE	18	0.046	0.004
Error	84	-	-
Coefficient of variation (%)	-	57.43	83.49
Overall mean	-	37.40	1.396

Table S2. Summary of analysis of variance (ANOVA) and probability values (*P*) for plant height, stem diameter, leaf number, fresh weight, and dry weight of biomass sorghum plants subjected to different silicon doses with and without *Melanaphis sorghi* infestation.

Source of Variation	Degrees of Freedom	Height	Stem Diameter	Leaf Number	Fresh Weight	Dry Weight
Silicon dose	3	0.070	0.840	0.274	0.006	0.139
Aphid infestation	1	0.006	0.528	0.022	0.002	0.098
Dose × Infestation	3	0.294	0.134	0.192	0.083	0.036
Error	16	-	-	-	-	-
Coefficient of variation (%)	-	14.05	15.50	12.73	22.76	29.01
Overall mean	-	135.175	9.596	8.979	94.530	13.147

Table S3. Summary of analysis of variance (ANOVA) and probability values (*P*) for lignin, cellulose, hemicellulose, and calorific value of biomass sorghum plants subjected to different silicon doses with and without *Melanaphis sorghi* infestation.

Source of Variation	Degrees of Freedom	Lignin	Cellulose	Hemicellulose	Calorific Value
Silicon dose	3	0.008	0.002	<0.001	0.245
Aphid infestation	1	<0.001	0.503	<0.001	0.015
Dose × Infestation	3	0.206	0.018	0.272	0.112
Error	16	-	-	-	-
Coefficient of variation (%)	-	7.47	9.11	4.70	2.71
Overall mean	-	3.983	33.641	28.976	16.023

Table S4. Summary of analysis of variance (ANOVA) and probability values (*P*) for nitrogen, phosphorus, potassium, calcium, magnesium, and sulfur in biomass sorghum plants subjected to different silicon doses with and without *Melanaphis sorghi* infestation.

Source of Variation	Degrees of Freedom	Nitrogen	Phosphorus	Potassium	Calcium	Magnesium	Sulfur
Silicon dose	3	<0.001	0.003	<0.001	<0.001	0.825	0.511
Aphid infestation	1	0.009	0.046	0.287	0.144	0.015	0.481
Dose × Infestation	3	0.959	0.183	0.887	0.447	0.799	0.269
Error	16	-	-	-	-	-	-
Coefficient of variation (%)	-	12.35	23.28	23.20	16.06	20.36	15.30
Overall mean	-	21.121	1.900	16.208	4.506	2.544	1.108

Table S5. Summary of analysis of variance (ANOVA) and probability values (*P*) for boron, copper, iron, manganese, zinc, and silicon in biomass sorghum plants subjected to different silicon doses with and without *Melanaphis sorghi* infestation.

Source of Variation	Degrees of Freedom	Boron	Copper	Iron	Manganese	Zinc	Silicon
Silicon dose	3	0.347	0.525	0.286	0.671	0.065	0.012
Aphid infestation	1	0.722	0.156	0.096	0.727	0.023	0.367
Dose × Infestation	3	0.107	0.749	0.277	0.001	0.008	0.652
Error	16	-	-	-	-	-	-
Coefficient of variation (%)	-	31.21	81.66	117.15	25.30	18.32	164.75
Overall mean	-	14.315	3.322	143.403	71.189	18.294	1395.497
